# Potential sustained benefits of early targeted panretinal photocoagulation in combination with anti-VEGF in macular edema secondary to retinal vein occlusion: 48-month results of a retrospective comparative study

**DOI:** 10.1186/s12886-025-04496-9

**Published:** 2025-11-13

**Authors:** Adnan Kilani, Koray Bayhan, Efstathios Vounotrypidis, Denise Vogt, Almut Bindewald-Wittich, Benjamin Mayer, Christian Enders, Armin Wolf

**Affiliations:** 1 Department of Ophthalmology, Ulm University Hospital, Prittwitzstraße 43, 89075 Ulm, Germany; 2 Institute of Epidemiology and Medical Biometry, Ulm University, Schwabstraße 13, 89075 Ulm, Germany; 3 Ophthalmology Consultants Werdenfels, Garmisch-Partenkirchen, Germany

**Keywords:** Anti-VEGF, Early targeted PRP, Ischemic index, Retinal vein occlusion, Macular edema

## Abstract

**Purpose:**

To evaluate the long-term benefit of early targeted panretinal photocoagulation (PRP) combined with anti-VEGF therapy (IVL group) versus anti-VEGF monotherapy (IV group) in treatment-naïve eyes with macular edema (ME) secondary to ischemic RVO.

**Methods:**

A retrospective analysis of 143 patients (85 IVL, 58 IV) with ischemic RVO. Baseline ischemic index (IsI), central retinal thickness (CRT), best-corrected visual acuity (BCVA), and age were adjusted.

**Results:**

Over 48 months, the IVL group showed a reduction in mean CRT from 475.6 ± 117.3 μm to 282.0 ± 69.5 μm and improved BCVA from 0.61 ± 0.34 LogMAR to 0.44 ± 0.34 LogMAR. The IV group demonstrated CRT reduction from 479.4 ± 135.5 μm to 340.9 ± 127.3 μm and BCVA improvement from 0.58 ± 0.32 LogMAR to 0.50 ± 0.43 LogMAR. The IVL group received 26.0 ± 8.6 intravitreal anti-VEGF treatments (IVT), compared to 25.5 ± 6.2 IVT in the IV group.

**Conclusions:**

The IVL group exhibited a trend toward better treatment response, particularly in patients with severe retinal ischemia, though findings were not statistically significant. Baseline IsI quantification is recommended for optimal RVO management.

**Supplementary Information:**

The online version contains supplementary material available at 10.1186/s12886-025-04496-9.

## Key messages

*What is known*:

- The benefit of adjuvant targeted panretinal photocoagulation (PRP) combined with intravitreal anti-VEGF treatment for macular edema (ME) secondary to ischemic retinal vein occlusion (RVO) remains controversial.
- Limited evidence suggests that early, targeted PRP of the ischemic peripheral retina may improve visual acuity (VA) and ME response to anti-VEGF treatment.

*What is new*:

- This is the first study to report long-term outcomes of combined early targeted PRP and anti-VEGF therapy versus anti-VEGF monotherapy in 143 eyes with ischemic RVO.
- Combined therapy showed a trend toward improved anatomical outcomes, particularly in patients with baseline ischemic index (IsI) ≥ 15%.
- Early PRP did not reduce the treatment burden, including the number of anti-VEGF injections.
- Quantifying IsI at baseline is crucial for assessing retinal ischemia severity in RVO.

**Supplementary Information:**

The online version contains supplementary material available at 10.1186/s12886-025-04496-9.

## Introduction

Retinal vein occlusion (RVO) is the second most common retinal vascular disorder, often leading to ME and visual deterioration [1, 2]. VEGF (vascular endothelial growth factor) is considered as a major mediator for ME in RVO [3]. Intravitreal injections with anti-VEGF agents or steroids are established as the gold standard, while focal laser has historically been the most effective treatment especially for branch retinal vein occlusion (BRVO) [1, 4–7].

However, some patients respond poorly to intensive anti-VEGF monotherapy, despite advances in imaging and new established anti-VEGF regimens [3]. Published data suggest that peripheral retinal nonperfusion plays an important role in the upregulation of VEGF production during retinal vascular diseases, with consequent effects on retinal neovascularization and ME [8–12]. Furthermore, the area of peripheral ischemic retina appears to correlate with the severity and recurrence of ME [9, 11, 13]. Some studies have demonstrated a better treatment response with lower recurrence rate of ME with additional early targeted panretinal photocoagulation (PRP) [14, 15]. These findings are supported by animal RVO models, which demonstrated intravitreal VEGF suppression and beneficial effects on ME after laser photocoagulation of the ischemic retinal area [12].

The benefit of early adjuvant PRP in these cases is still under discussion. Recent studies recommend the assessment of peripheral nonperfused retinal areas using widefield fluorescein angiography (FA) to evaluate the ischemic index (IsI), which represents the ratio of the area of nonperfused retina as a percentage of the total visible retina [10, 11]. Currently, PRP is only used for treatment of neovascular complications associated with RVO or prophylactically in case of extensive retinal ischemia [1].

This study evaluates the long-term efficacy of combined early PRP and anti-VEGF therapy (IVL group) versus anti-VEGF monotherapy (IV group) in treatment-naïve eyes with ME secondary to ischemic RVO.

## Materials and methods

### Patients and methods

This retrospective, comparative, cohort study was conducted at the Department of Ophthalmology, Ulm University Hospital, Germany and approved by the local ethics committee (application number 277/21). All procedures performed in this study were in accordance with the ethical standards of the institutional research committee and with the 1964 Helsinki declaration and its subsequent amendments or comparable ethical standards.

**Fig. 1:**
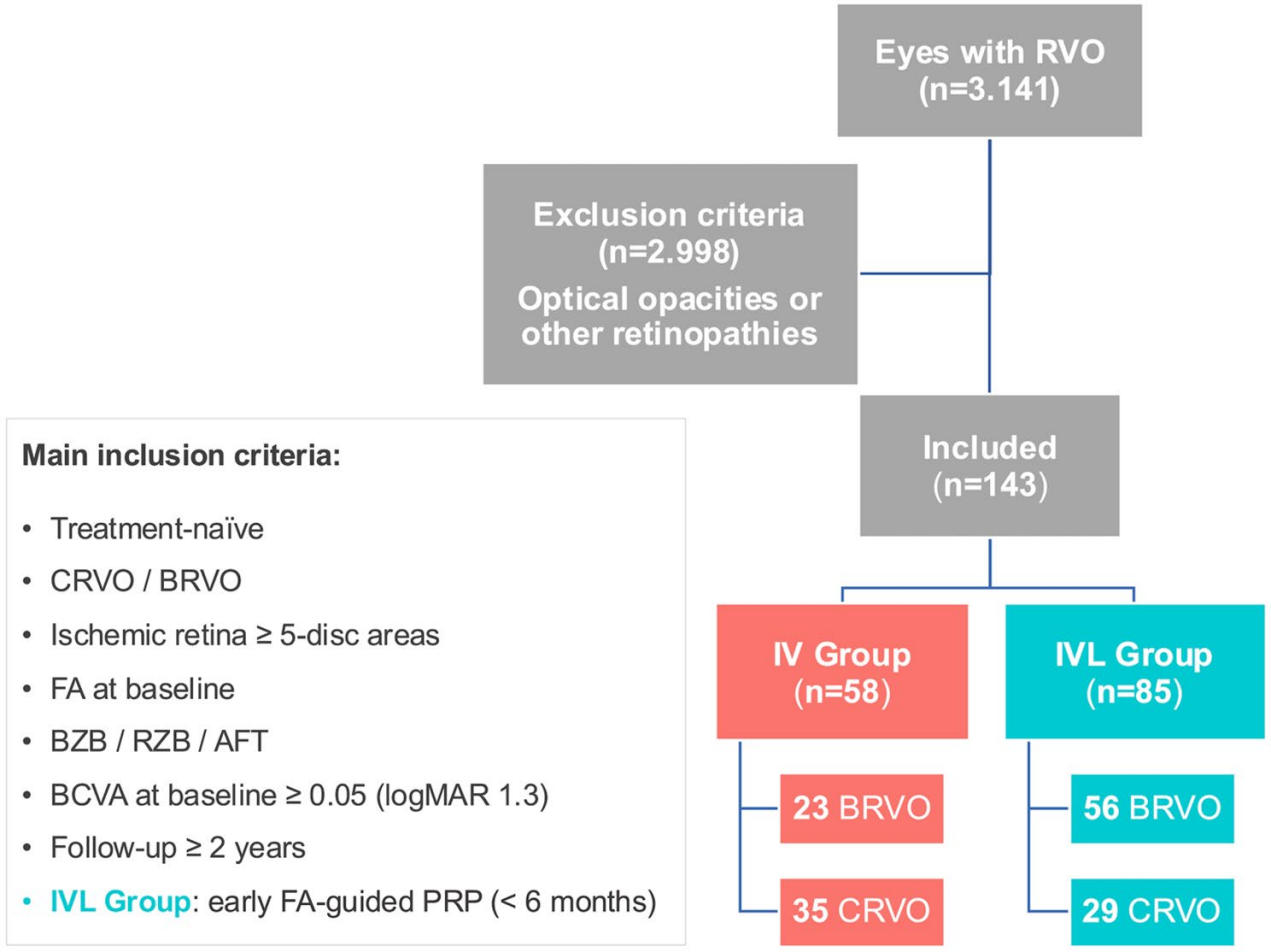
Flow Chart illustrating the selection process. Abbreviations: AFT: Aflibercept 2 mg; BCVA: best-corrected visual acuity; BRVO: Branch Retinal Vein Occlusion; BZB: Bevacizumab; CRVO: Central retinal vein occlusion; FA: fluorescein angiography; PRP: panretinal photocoagulation; RZB: Ranibizumab

A total of 3,141 eyes with RVO were treated in our department since 2010 (Fig. 1).

Main inclusion criteria were treatment-naïve patients with ME secondary to recently diagnosed ischemic RVO, including BRVO or central retinal vein occlusion (CRVO) subtypes, confirmed within 6 months prior to screening. Eligible patients also had peripheral retinal ischemia of ≥ 5-disc areas (BRVO) or ≥ 10-disc areas (CRVO), as detected by ultra-widefield fluorescein angiography (FA) or standard 7-field FA with peripheral imaging. Ischemic CRVO was defined per the Central Vein Occlusion Study (CVOS) as ≥ 10-disc areas of nonperfusion, while ischemic BRVO was defined per the Branch Vein Occlusion Study (BVOS) as ≥ 5-disc areas of nonperfusion [1, 9–11].

Exclusion criteria included diagnosis of ocular disease other than RVO that may contribute to ME (i.e., wet age-related macular degeneration, diabetic retinopathy, choroidal neovascularization, or uveitis), iris or retinal neovascularization at baseline, follow-up shorter than 2 years, baseline best-corrected visual acuity (BCVA) worse than 1.3 LogMAR, uncontrolled glaucoma, other vitreoretinal pathology (i.e., vitreomacular traction, epiretinal membrane or macular hole on SD-OCT), previous vitreoretinal surgery, a steroid intravitreal injection or implant at any time point, macular laser photocoagulation at any time point, significant media opacities (i.e., cataract, vitreous hemorrhage), or image artefacts that precluded photographic evaluation of ME and peripheral retinal capillary perfusion status.

The study analyzed 143 eyes with ischemic RVO according to the SCORE study, including hemi-central retinal vein occlusion (hemi-CRVO) as a subtype of CRVO. Treatment options were: monotherapy consisting of guideline-directed anti-VEGF treatment (IV group) or combination therapy with additional early targeted PRP (IVL group).

The rationale for the study stems from a significant change in the treatment protocol for RVO at the Department of Ophthalmology, Ulm University Hospital, Germany.

Since 2020, PRP in the ischemic retinal area has been discontinued and anti-VEGF monotherapy became our institutional standard of care. All ischemic RVO diagnosed in 2020 received the new standard treatment protocol, based on guideline-recommended initiation of anti-VEGF therapy for RVO, has been rigorously implemented. This shift prompted a retrospective comparison of the outcomes between the two treatment groups to evaluate the efficacy and implications of the updated approach.

**Fig. 2:**
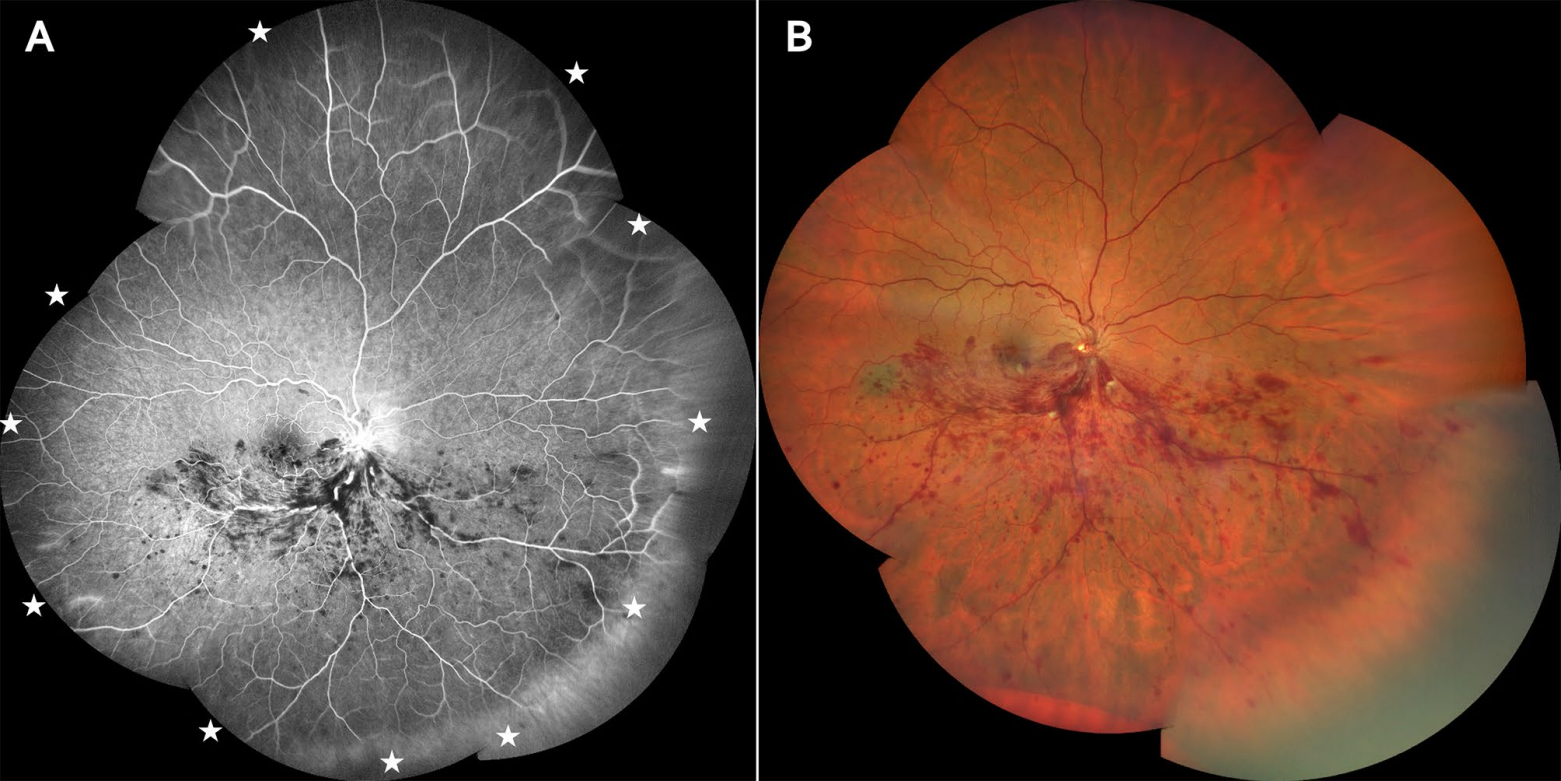
(**A**) Multiview composite fluorescein angiography image prior to panretinal photocoagulation, with the white star indicating the area of retinal ischemia. (**B**) Multiview composite color fundus photograph prior to panretinal photocoagulation

**Fig. 3:**
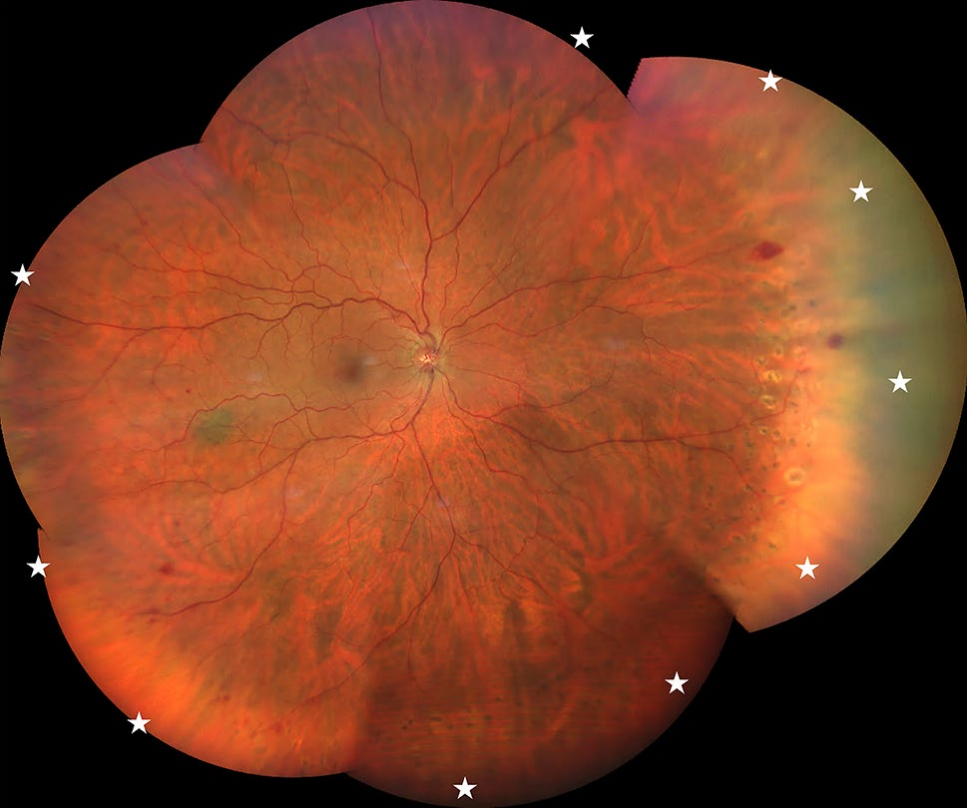
Multiview composite color fundus photograph after panretinal photocoagulation, with the white star indicating the area treated based on fluorescein angiography guidance

During at least two years of follow-up, both treatment groups received the anti-VEGF agents available at that time, including bevacizumab (BZB), ranibizumab (RZB), and aflibercept 2 mg (AFT). Since 2020, patients received intravitreal injections according to treat and extend (TAE) regimen, whereas before 2020 they received injections according to pro re nata (PRN) regimen as shown in Table 1. The adjustment of the treatment interval according to TAE or PRN regimen was based on the presence of intraretinal fluid (IRF) with a central retinal thickness (CRT) ≥ 300 μm or, in rare cases, at the physician’s discretion. In addition, anti-VEGF agents were switched in cases where patients demonstrated a suboptimal treatment response, as characterized by persistent IRF with CRT thickness ≥ 280 μm after administration of at least six injections. Early FA-guided PRP was performed exclusively in the IVL group within the first 6 months after screening. PRP was routinely performed under FA guidance using small laser spots (100–200 μm), moderate power (100–200 mW), and a pulse duration of 100 ms, as illustrated in Figs. 2 and 3. To ensure a focused evaluation of early FA-guided PRP, patients who received PRP beyond the 6-month window were excluded from the study. In contrast, anti-VEGF injections were initiated at the time of the patient’s initial presentation. As previously described, patients received between 1 and 5 anti-VEGF injections before undergoing their first PRP session. The flowchart in Fig. 1 provides an overview of the study design and illustrates the selection process.

**Table 1:** Patient demographic characteristics and baseline ocular characteristics

Characteristics	IV Group (*n* = 58)	IVL Group (*n* = 85)
Age, mean (SD), y	66.7 (± 12.0)	67.2 (± 11.3)
male : female (n)	26 : 32	44 : 41
BRVO : CRVO (n)	23 : 35	56 : 29
IsI, mean (SD), %	7.5 (± 5.4)	8.6 (± 7.1)
TAE : PRN	13 : 45	7 : 78
BZB : RZB : AFT	34 : 15 : 9	46 : 24 : 15
CRT, mean (SD), µm	479.4 (± 135.5)	475.6 (± 117.3)
BCVA, mean (SD), LogMAR	0.58 (± 0.32)	0.61 (± 0.34)

Abbreviations: AFT: Aflibercept; BCVA: best-corrected visual acuity; BRVO: branch retinal vein occlusion; BZB: Bevacizumab; CRVO: central retinal vein occlusion; CRT: central retinal thickness; IsI: ischemic index; PRN: pro re nata treatment regimen; RVO: retinal vein occlusion; RZB: Ranibizumab; TAE: treat and extend treatment regimen; SD: standard deviation

All patients underwent comprehensive ophthalmologic examination including medical history, best-corrected visual acuity (BCVA), slit lamp, fundus examination, SD-OCT imaging (Cirrus OCT, Carl Zeiss Meditec Inc. or Spectralis OCT, Heidelberg Engineering Inc.) and FA with peripheral FA images (Zeiss FF450PlusIR Fundus Camera, Carl Zeiss Meditec Inc.; Clarus, Carl Zeiss Meditec Inc. or Spectralis HRA + OCT SLO, Heidelberg Engineering Inc.). To assess retinal vascular perfusion status, standard multifield FA with 7-view photography was performed during the first 6 months, once retinal hemorrhages resolved. Two graders (AK, EV) selected one or more images from each angiogram series after complete arteriovenous filling, evaluating them for nonperfusion as previously described [16].

IsI was assessed using established methods [9, 11]. The image with the widest field of view and optimal clarity was selected, with adjustments allowed for contrast, brightness, and gamma. IsI was calculated as the percentage of nonperfused retinal area relative to the total gradable retina, based on ultra-widefield fluorescein angiography (UWFA) or multi-field fluorescein angiography (FA). Nonperfused areas were segmented using either RegionFinder (Heidelberg Engineering Inc.) or FIJI/ImageJ. The ratio of nonperfused to total gradable pixels was multiplied by 100 to yield the IsI (%). Moreover, no standardized follow-up FAs were performed for therapy monitoring; FA was only repeated in cases of recurrent RVO or clinical deterioration.

### Endpoints and statistical analysis

**Fig. 4:**
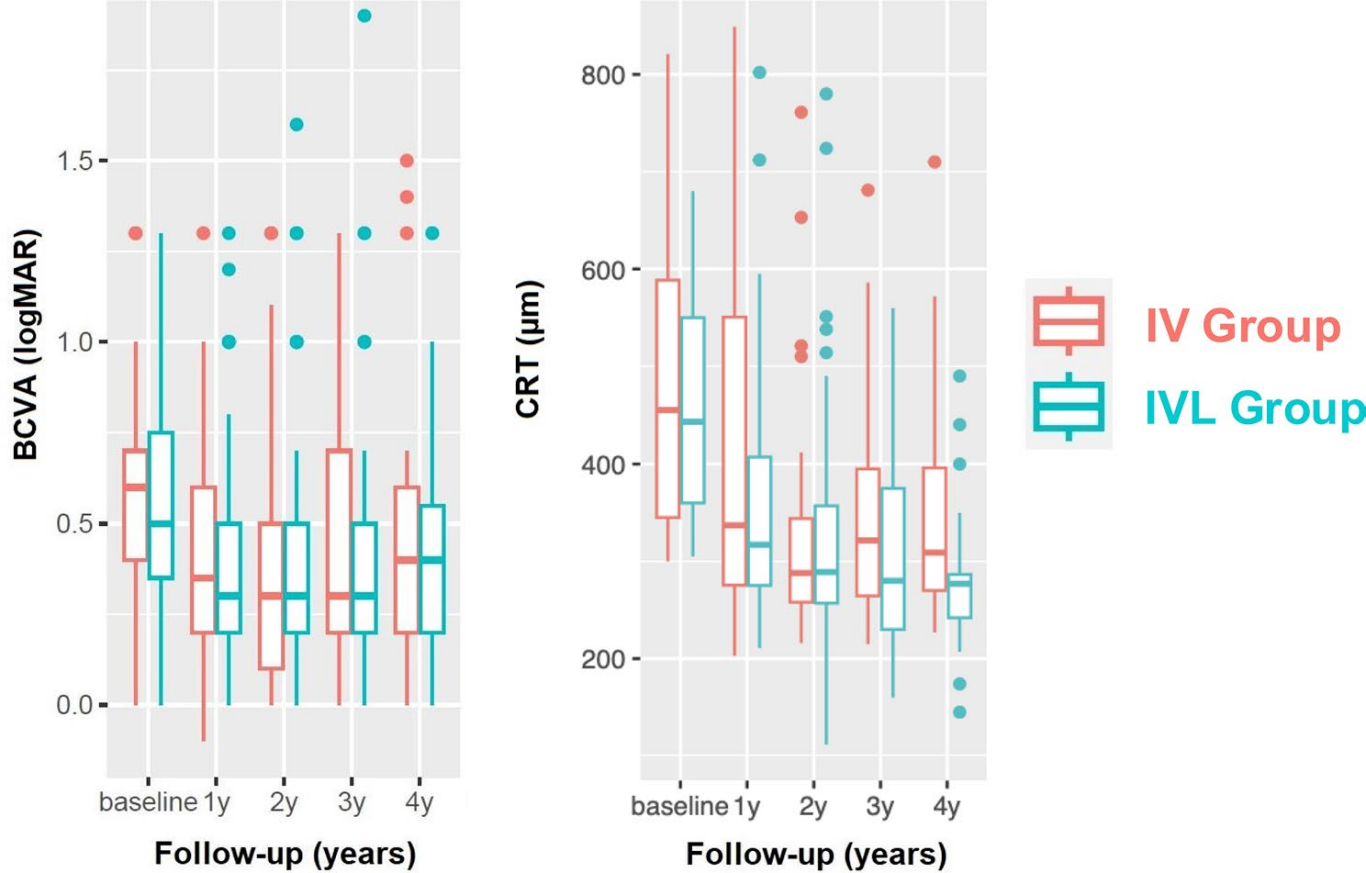
Boxplots demonstrating a treatment-IsI interaction regarding mean BCVA and CRT change 48 months of follow-up. Abbreviations: BCVA: best-corrected visual acuity; CRT: central retinal thickness; IsI: ischemic index

Outcome measures included changes in mean BCVA, mean CRT (Fig. 4), and mean number of intravitreal anti-VEGF treatments (IVTs) during follow-up, as detailed in Tables 2 and 3. Descriptive statistics were calculated using arithmetic mean and standard deviation (SD) for continuous variables and frequencies for categorical variables. Due to potential deviations from normality in CRT, BCVA, and IVT comparisons, non-parametric Mann-Whitney U tests were employed when appropriate. To address repeated measurements, random intercept mixed linear regression models were applied, incorporating group status (IVL vs. IV), time of measurement, and their interaction as fixed predictors. A dichotomous covariate for ischemia severity (IsI ≥ 10%, IsI ≥ 15%, and IsI ≥ 20%) was included as a potential confounder and evaluated for treatment interaction. Statistical significance was set at *p* < 0.05 (Table 4). Both groups were adjusted for baseline IsI, CRT, BCVA, and age.

**Table 2:** 24-Months results (Compared to Baseline)

Characteristics	IV Group (*n* = 58)	IVL Group (*n* = 85)	*P*-value
CRT, mean (SD), µm	336.9 (± 127.8)	317.3 (± 120.4)	0.309
Change in CRT, mean (SD), µm	-137.7 (± 157.8)	-159.9 (± 172.9)	0.327
BCVA, mean (SD), LogMAR	0.39 (± 0.41)	0.46 (± 0.37)	0.093
Change in BCVA, mean (SD), LogMAR	-0.20 (± 0.44)	-0.16 (± 0.33)	0.349
Total No. of IVT at month 24, mean (SD)	12.0 (± 4.7)	13.4 (± 4.8)	0.072

Abbreviations: BCVA: best-corrected visual acuity; CRT: central retinal thickness; IVT: intravitreal treatments; SD: standard deviation

**Table 3:** 48-Months results (Compared to Baseline)

Characteristics	IV Group (*n* = 19)	IVL Group (*n* = 38)	*P*-value
CRT, mean (SD), µm	340.9 (± 127.3)	282.0 (± 69.5)	0.181
Change in CRT, mean (SD), µm	-178.0 (± 175.3)	-193.4 (± 133.0)	0.874
BCVA, mean (SD), LogMAR	0.50 (± 0.43)	0.44 (± 0.34)	0.756
Change in BCVA, mean (SD), LogMAR	-0.13 (± 0.49)	-0.25 (± 0.51)	0.261
Total No. of IVT at month 48, mean (SD)	25.5 (± 6.2)	26.0 (± 8.6)	0.644

Abbreviations: BCVA: best-corrected visual acuity; CRT: central retinal thickness; IVT: intravitreal treatments; SD: standard deviation

**Table 4:** Longitudinal results following a mixed model approach

Characteristics	IVL Group vs. IV Group Estimate (SE)	*P*-value	*P*_adj_10_	*P*_adj_15_	*P*_adj_20_
CRT, µm	-23.81 (14.58)	0.105	0.081	0.054	0.064
BCVA, LogMAR	0.038 (0.053)	0.474	0.515	0.584	0.902
Total No. of IVT	1.384 (0.921)	0.135	0.291	0.298	0.280

Abbreviations: BCVA: best-corrected visual acuity; CRT: central retinal thickness; IVT: intravitreal treatments; SE: standard error

Estimates and SE refer to a time-adjusted mixed model; adjusted P-values P_adj_10_, P_adj_15,_ and P_adj_20_ of the estimated IVL Group vs. IV Group difference refer to respective models in which a dichotomous covariate indicating IsI ≥ 10%, IsI ≥ 15% and IsI ≥ 20% was considered as an additional confounding variable

## Results

### Demographic and baseline characteristics

Between 2010 and 2021, 85 patients (44 men [52%], 41 women [48%]; mean age 67.2 ± 11.3 years) in the IVL group and 58 patients (26 men [45%], 32 women [55%]; mean age 66.7 ± 12.0 years) in the IV group were included. Baseline characteristics were similar between groups (Table 1). Mean baseline BCVA was 0.61 ± 0.34 LogMAR in the IVL group and 0.58 ± 0.32 LogMAR in the IV group. Mean CRT was 475.6 ± 117.3 μm in the IVL group and 479.4 ± 135.5 μm in the IV group. Mean IsI was 8.6 ± 7.1% in the IVL group and 7.5 ± 5.4% in the IV group.

Thirteen patients (22.4%) in the IV group and seven patients (8.2%) in the IVL group received IVT according to the TAE regimen. Follow-up from 24 to 48 months was completed by 38 patients (44.7%) in the IVL group and 19 patients (32.8%) in the IV group. At baseline, 58.6% of the IV group and 54.1% of the IVL group received IVT with bevacizumab (BZB). Baseline demographic and ocular characteristics were similar between the two groups, as detailed in Table 1.

### Anatomic outcomes

At 24 months, mean CRT decreased to 317.3 ± 120.4 μm in the IVL group and 336.9 ± 127.8 μm in the IV group (*P* = 0.309). The mean CRT change from baseline to 24 months was − 159.9 ± 172.9 μm in the IVL group and − 137.7 ± 157.8 μm in the IV group (*P* = 0.327).

At 48 months, mean CRT further improved to 282.0 ± 69.5 μm in the IVL group and 340.9 ± 127.3 μm in the IV group (*P* = 0.181). The mean CRT change from baseline to 48 months was − 193.4 ± 133.0 μm in the IVL group and − 178.0 ± 175.3 μm in the IV group (*P* = 0.874). Mixed linear regression models showed no significant treatment group × time interaction (*P* = 0.105). However, a trend toward greater fluid reduction was observed in the IVL group, particularly in patients with IsI ≥ 15% (*P* = 0.054) (Table 4).

### Functional outcomes

At 24 months, mean BCVA improved to 0.46 ± 0.37 LogMAR in the IVL group and 0.39 ± 0.41 LogMAR in the IV group (*P* = 0.093). The mean BCVA gain from baseline to 24 months was − 0.16 ± 0.33 LogMAR in the IVL group and − 0.20 ± 0.44 LogMAR in the IV group (*P* = 0.349). At 48 months, mean BCVA was 0.44 ± 0.34 LogMAR in the IVL group and 0.50 ± 0.43 LogMAR in the IV group (*P* = 0.756). Mixed linear regression models showed no significant treatment group × time interaction (*P* = 0.474), even after adjusting for IsI (Table 4).

### Mean numbers of injections

At 24 months, the mean number of IVTs was 13.4 ± 4.8 in the IVL group and 12.0 ± 4.7 in the IV group (*P* = 0.072). At 48 months, the IVL group received a mean of 26.0 ± 8.6 IVTs, compared to 25.5 ± 6.2 IVTs in the IV group (*P* = 0.644). Mixed linear regression models showed no significant treatment group × time interaction (*P* = 0.135), regardless of IsI adjustment (Table 4).

## Discussion

This study is among the first to investigate the long-term outcomes of combined anti-VEGF therapy and early targeted PRP compared to anti-VEGF monotherapy in ME secondary to ischemic RVO. While our findings suggest a potential benefit of the combined approach, particularly in eyes with a baseline IsI ≥ 15%, these trends did not achieve statistical significance in terms of visual acuity (VA) or treatment burden. This highlights the complexity and heterogeneity of RVO and its variable response to treatment.

The role of early PRP in managing secondary ME in ischemic RVO remains a subject of debate [4, 17–19]. To date, only a limited number of studies have demonstrated that early, targeted PRP of the ischemic peripheral retina, in conjunction with IVT, may improve VA and enhance the response of ME to IVT [14, 15]. However, the lack of superiority of combined therapy over anti-VEGF monotherapy in several studies may be attributed to their design, which often included ME cases regardless of perfusion status or severity of retinal ischemia, or employed inconsistent timing of PRP, sometimes exceeding six months post-diagnosis [17–19]. Our study did not confirm a definitive benefit of early targeted PRP, underscoring the need for further investigation. Additionally, the observation that combined therapy did not reduce the number of IVT injections aligns with some, but not all, prior studies [18, 20, 21].

Notably, patients with a baseline IsI ≥ 15% showed in our cohort a more pronounced reduction in CRT with combined therapy. This supports the hypothesis that untreated extensive non-perfused areas may drive VEGF-mediated ME recurrence [9, 11, 13]. It is plausible that in patients with larger areas of peripheral nonperfusion, PRP may exert a greater impact on controlling ME and improving VA compared to those with smaller areas of nonperfusion [10]. Here it was hypothesized that the extent of peripheral ischemia in RVO may be the driving force in rebound ME after IVT by affecting the levels of intravitreal VEGF, and in turn, modulate the severity of ME and its response to therapy [10]. Our results support this hypothesis and suggest that untreated extensive non-perfused areas may be the source of production of biochemical mediators that promote neovascularization and ME [9, 11]. While prior studies have identified an IsI ≥ 35% as a critical angiographic criterion for classifying CRVO as ischemic within the first year of follow-up [22], our study found a lower threshold of IsI ≥ 15% at baseline to be significant.

This study has several limitations. First, its retrospective design and the lack of statistical significance in many comparisons limit the strength of the conclusions. Although we observed a trend toward greater benefit from early FA-guided PRP in severe ischemic RVO, this finding should be interpreted with caution. In early RVO, FA can be challenging due to hemorrhage obscuring the view, which reduces its diagnostic utility in routine practice.

Second, although a standardized laser protocol was applied, the number of laser spots or sessions per eye was not consistently documented and may not accurately represent treatment intensity, as not all attempted burns achieve effective delivery. For this reason, we focused on detailed procedural parameters rather than absolute spot counts.

Third, BRVO and CRVO were initially analyzed separately; however, no significant interaction between treatment effect and RVO subtype was identified, even when stratified by ischemia severity (IsI ≥ 10%, ≥ 15%, or ≥ 20%). Despite their pathophysiological differences, both conditions share complications such as ME and ischemia, and are often managed similarly. To enhance statistical power and interpretability, we combined BRVO and CRVO cases into a single RVO cohort for the final analysis. This approach revealed a near-significant difference in CRT reduction at 4 years in the IVL group when baseline IsI ≥ 15% (Table 4). Nonetheless, group imbalances may have influenced these results, as CRVO accounted for 60% of the IV group but only 34% of the IVL group.

Fourth, visual field (VF) changes - an important potential side effect of PRP - were not evaluated because VF testing was not routinely performed and was therefore unavailable for this retrospective analysis. In clinical practice, VF testing is rarely conducted in RVO patients. Importantly, small-spot, moderate-energy PRP was used in our cohort, and ischemic RVO itself can contribute to VF loss. As reported by Lee et al., untreated retinal ischemia may cause progressive VF deterioration, whereas PRP - despite initial VF reduction - may ultimately help preserve VF, particularly in high-risk proliferative diabetic retinopathy [22].

Finally, heterogeneity in ischemic severity and a high dropout rate - likely exacerbated by the COVID-19 pandemic and the change in anti-VEGF treatment regimen from PRN to TAE after 2020 (55.3% in the IVL group, 67.2% in the IV group at 4 years) - may have further limited statistical power and influenced outcomes. These factors underscore the need for future prospective studies with larger, balanced treatment groups and standardized follow-up.

Despite its limitations, our study offers several strengths, including a long-term follow-up period of 48 months, a relatively large sample size, and a homogeneous study population achieved through careful patient selection and matched treatment groups. Baseline IsI values were comparable between groups, ensuring a similar prognosis for treatment outcomes. However, future prospective studies are necessary to establish clinically significant differences and perform non-inferiority analyses to further validate these findings.

In conclusion, while our study suggests a potential trend toward improved anatomical outcomes with combined early PRP and anti-VEGF therapy, particularly in severe ischemic RVO, this benefit did not reach statistical significance and did not reduce the treatment burden. Quantifying IsI at baseline remains crucial for guiding treatment decisions. Future prospective studies are warranted to validate these findings and refine therapeutic strategies for ME secondary to RVO.

## Supplementary Information

Below is the link to the electronic supplementary material.

Supplementary Material 1

## Data Availability

All data supporting the findings of this study are included within the article. Detailed patient data, which are sensitive in nature, are not publicly available but can be provided in anonymized form by the corresponding author upon reasonable request.

## Abbreviations

AFT: Aflibercept 2 mg
BCVA: Best-corrected visual acuity
BRVO: Branch retinal vein occlusion
BZB: Bevacizumab
CRVO: Central retinal vein occlusion
CRT: Central retinal thickness
FA: Fluorescein angiography
IsI: Ischemic index
ME: Macular edema
PRN: Pro re nata treatment
PRP: Panretinal photocoagulation
RVO: Retinal vein occlusion
RZB: Ranibizumab
TAE: Treat and extend treatment
SD: Standard deviation
SE: Standard error
IV group: Anti-VEGF monotherapy group
IVL group: Early targeted PRP combined with anti-VEGF therapy
IVT: Intravitreal treatments
VF: Visual field
